# Trends in type 1 diabetes incidence between 2007 and 2023 and their association with SARS-CoV-2 infection in a population-based matched cohort study among individuals under 30 years old in Sweden

**DOI:** 10.1007/s00125-025-06540-1

**Published:** 2025-09-18

**Authors:** Dominik Dietler, Jonas Björk, Elsa Palmkvist, Annelie Carlsson

**Affiliations:** 1 Division of Occupational and Environmental Medicine, Lund University, Lund, Sweden; 2 Clinical Studies Sweden, Forum South, Skåne University Hospital, Lund, Sweden; 3 Department of Clinical Sciences, Department of Pediatrics, Skåne University Hospital, Lund, Sweden

**Keywords:** COVID-19, Epidemiology, Type 1 diabetes, Viral disease

## Abstract

**Aims/hypothesis:**

The incidence of type 1 diabetes increased during the pandemic in various countries. SARS-CoV-2 infections may trigger the development of type 1 diabetes, but the evidence is inconclusive. This study aimed to assess trends in type 1 diabetes incidence between 2007 and 2023, and to quantify the association between SARS-CoV-2 infections and the risk for developing type 1 diabetes.

**Methods:**

The study included all individuals under 30 years old registered in Sweden. Deviations in type 1 diabetes incidence from pre-pandemic trends (2007–2019) were assessed for each pandemic year (2020–2023) using Poisson regression. The effect of SARS-CoV-2 infections was assessed using Cox proportional hazards models in a cohort of infected individuals with five control individuals from the infection date of the case, matched by birth year, sex and region.

**Results:**

Compared with the predicted linear trend, type 1 diabetes incidence increased by 12% during 2021 (incidence rate ratio [IRR] 1.12; 95% CI 1.06, 1.19) and 9% during 2022 (IRR 1.09; 95% CI 1.02, 1.16), but reverted to pre-pandemic trends in 2023. Overall, the adjusted HR for developing type 1 diabetes after SARS-CoV-2 infection was 0.96 (95% CI 0.79, 1.16). Children between 5 and 10 years old were more likely to develop type 1 diabetes within the first 28 days after infection (HR 2.68; 95% CI 1.22, 5.89), although their hazard over the whole follow-up period was not increased.

**Conclusions/interpretation:**

Sweden, with its non-restrictive pandemic response, saw a transient increase in type 1 diabetes incidence that was only partially associated with SARS-CoV-2 infections. Other explanations should be investigated, including environmental and lifestyle factors.

**Graphical Abstract:**

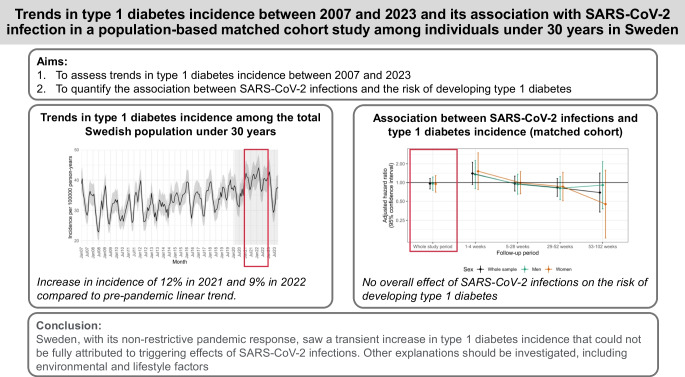

**Supplementary Information:**

The online version of this article (10.1007/s00125-025-06540-1) contains peer-reviewed but unedited supplementary material.


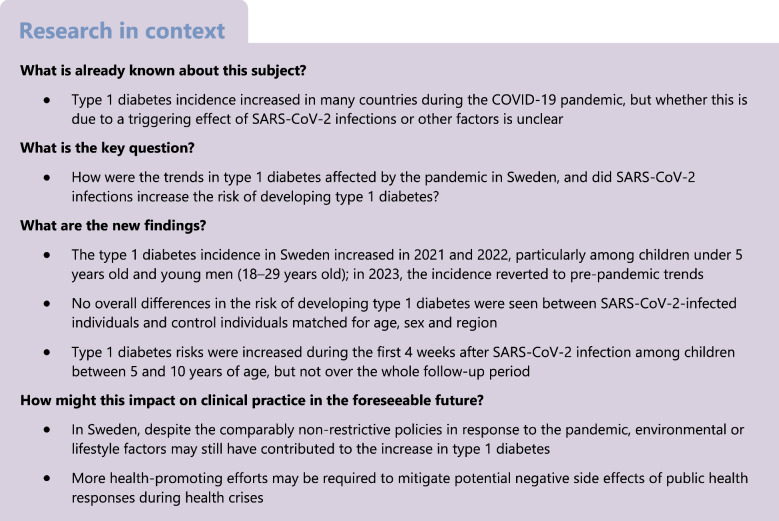


## Introduction

Type 1 diabetes is an autoimmune disease manifested by destruction of the beta cells in the pancreatic islet cells that produce insulin [1]. The aetiology of the disease is not fully understood, but both genetic and environmental factors, including viral infections, seem to play a major role in the development of type 1 diabetes [2–4]. In recent decades, the incidence of type 1 diabetes has increased globally [5–8]. A series of epidemiological studies have reported an increasing number of people with newly diagnosed type 1 diabetes during the COVID-19 pandemic [9–13]. However, it is still unclear whether this increase is directly linked to SARS-CoV-2 infections or changes in other environmental and behavioural factors during the pandemic. While some studies found an increased risk of developing type 1 diabetes [10, 14–17], or of developing islet autoantibodies after SARS-CoV-2 infection [18], other studies reported no association [19–22].

Sweden’s public health policies to manage the pandemic were mainly based on recommendations such as advice on social distancing, working from home and limiting gatherings, rather than implementing a strict lockdown as in other countries. Schools remained largely open, although there was an increased use of remote teaching approaches at secondary and tertiary level. At the same time, Sweden has some of the highest rates of type 1 diabetes incidence in the world [5, 8]. Hence, Sweden provides an interesting case study for disentangling the effects of SARS-CoV-2 infections from the effects of changing lifestyles during the pandemic. Therefore, the aims of this study were: (1) to analyse the temporal trends in type 1 diabetes between 2007 and 2023; and (2) to assess the effect of SARS-CoV-2 infections on the risk of developing type 1 diabetes among all individuals under 30 years old living in Sweden.

## Methods

### Study design and population

The source population for this study consisted of all individuals under 30 years old who were registered as living in Sweden at some point between 1990 and 2023. For the first aim, we created a dynamic population to study the trends in type 1 diabetes incidence between 2007 and 2023 (‘incidence population’) using individual-level data. To study the association between SARS-CoV-2 infection and the risk of new-onset type 1 diabetes, we created three matched cohorts. To ensure that complete information was available regarding SARS-CoV-2 infections, we excluded people who migrated during the pandemic by selecting only individuals who were registered before 1 February 2020 (the onset of the COVID-19 pandemic in Sweden) or were born during the pandemic. We also excluded individuals with a type 1 diabetes diagnosis prior to the pandemic. The matched cohorts differed in terms of their exposure definition. In the first cohort (‘individual infection cohort’), only individuals who themselves tested positive for SARS-CoV-2 between 1 February 2020 and 28 February 2022 were considered as infected. After February 2022, people with mild symptoms were no longer advised to get tested for SARS-CoV-2 in Sweden. Individuals who had an infection within the household more than 30 days prior to their positive test were excluded because they may have had an undetected infection previously. To estimate effects among the youngest age groups (for whom testing was less widespread in Sweden), we created a second cohort using the date of the first SARS-CoV-2 infection of any person living in the same household as a proxy case date (‘household infection cohort’). Only the first infection within the household was considered. In both cohorts, up to five control individuals who did not have any prior SARS-CoV-2 infection themselves or in their households were included, matched by case date (day of positive test), sex, exact birth year and regional statistical area (based on RegSo; division of Sweden into 3363 areas). Both cases and control individuals had to have been registered for at least 1 year before the case date to capture pre-existing type 1 diabetes.

A third cohort was used to perform a sensitivity analysis to test whether potential differences in testing behaviours affect the results. We used a test-negative design that included individuals who ordered a COVID-19 test through Sweden’s national health hotline (1177 hotline) (‘test-negative cohort’). Cases included individuals who tested positive for SARS-CoV-2 in such a test. We matched cases to up to five control individuals who ordered a test but were negative for SARS-CoV-2 within a 14-day window before and after the case date, based on birth year, sex and region. In all cohorts, individuals were followed up until the earliest of the date of death, emigration, type 1 diabetes diagnosis, their 30th birthday, the end of the study (28 February 2022) or (for control individuals only) having a positive SARS-CoV-2 test themselves or among a person living in the same household.

### Data sources

We used the comprehensive Swedish register infrastructure, which provides almost complete coverage of the entire population in Sweden linked through their unique personal identification number. To identify individuals diagnosed with diabetes, we triangulated data from the National Patient Register, the National Prescribed Drug Register and the Swedish National Diabetes Register. The first two registers are hosted by the National Board of Health and Welfare and include all diagnoses made during inpatient and specialised outpatient care visits, and all drug prescriptions in Sweden, respectively. The Swedish National Diabetes Register contains information on patients with diabetes, including the diabetes type and date of onset. Data on positive SARS-CoV-2 tests were available from the national reporting system for notifiable diseases (SmiNet) and data on COVID-19 vaccinations from the National Vaccination Register, both managed by the Public Health Agency of Sweden. Socio-demographic data (i.e. age, sex, residence, household and family composition), as well as information on immigration and emigration history, were taken from the Total Population Register maintained by Statistics Sweden. The same agency also provided data on household disposable income through the ‘longitudinal integrated database for health insurance and labour market studies’ (LISA), which includes information on individual incomes from various sources, such as employment, pension benefits or social insurances. For the sensitivity analyses, data on ordered COVID-19 tests were retrieved from Inera, which manages the data collected through the national health hotline (1177). The data included a subset of the positive cases reported to SmiNet. In addition, they included individual-level information on COVID-19 tests that were negative.

### Variables

The exposure variable was confirmed SARS-CoV-2 infection of the individual (‘individual infection cohort’) or of any household member, including the individual (‘household infection cohort’). Only the first infection or the first infection within the household was considered, respectively. The outcome of the study was onset of type 1 diabetes, defined as the earlier of the date of first diagnosis of type 1 diabetes (ICD-10 code E10) in the patient registers or the listed date of onset of type 1 diabetes in the Swedish National Diabetes Register.

We use the family history of type 1 diabetes or type 2 diabetes prior to the case date as an adjustment variable. Patients with type 2 diabetes were identified from the patient registers (ICD-10 code E11) and the National Prescribed Drug Register (prescription of diabetes medication; ATC classification code A10). Only prescriptions of diabetes medication among individuals over the age of 35 years old were considered as an indication of type 2 diabetes. Further covariates included vaccination status and household disposable income. Individuals were considered as vaccinated if they had received a second vaccine dose before the case date. Household disposable income was estimated by Statistics Sweden, correcting the total household income using age-specific weights for household members. Missing household income values were assigned the median value for the total population.

### Statistical analyses

For each month between January 2007 and December 2023, we calculated the incidence of type 1 diabetes using 3-month rolling time windows. We used individual-level data to calculate the total time at risk among the population. Individuals were considered as being at risk of developing type 1 diabetes starting from the latest date among birth, immigration and the start of the study (1 January 2007) and ending with the earliest among death, emigration, type 1 diabetes diagnosis, their 30th birthday, and the end of the study (31 December 2023). Individuals who repeatedly immigrated could re-enter the cohort. For 2023, no data on births, deaths and migration were available, so we estimated the number of person-years based on the linear trend in the two previous years.

Deviations in the yearly incidence during the pandemic years were quantified using Poisson regression. The models were robust against over-dispersion, returning similar results to those for negative binomial regression models. The incidence in the pre-pandemic years (i.e. 2007–2019) was used as a reference. We incorporated a linear term for the calendar year to adjust for the long-term trend in type 1 diabetes incidence. The models were further adjusted for age and sex to account for fluctuations in population composition.

To assess the association between SARS-CoV-2 infection and the risk of new-onset type 1 diabetes, we used Cox proportional hazards regression. The first day of the follow-up period (i.e. the day of a confirmed positive COVID-19 test of the case) was excluded to remove coincidentally identified SARS-CoV-2 infections during type 1 diabetes-associated healthcare visits. We adjusted for the natural logarithm of the disposable household income, vaccination status at the day of infection, and family history of type 1 diabetes and type 2 diabetes. To account for individual-level clustering, robust standard errors were calculated using the Huber sandwich estimator in all models.

**Fig. 1:**
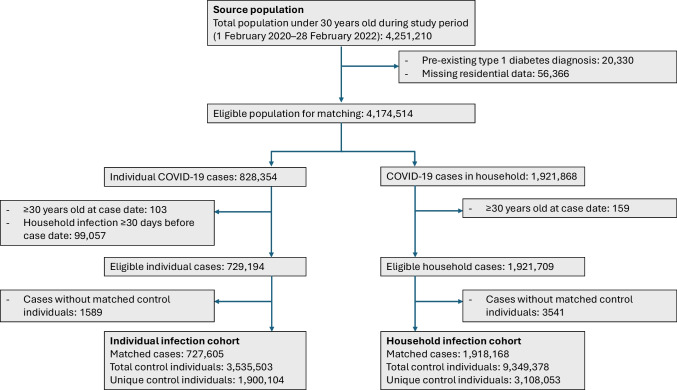
Flow chart showing the selection of cases and control individuals in the individual and household infection cohorts

**Fig. 2:**
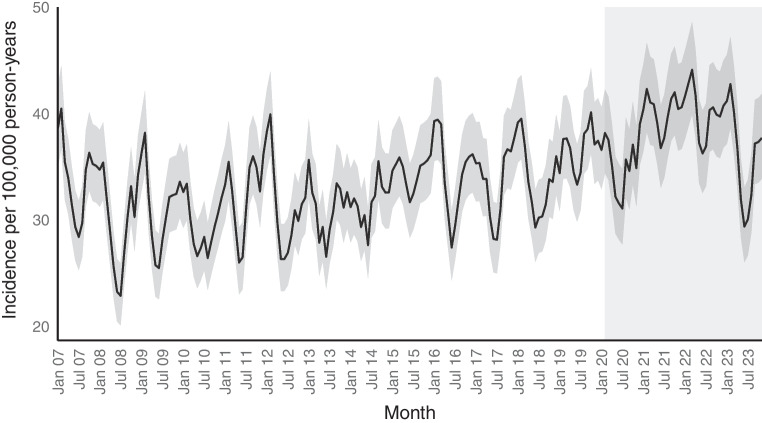
Incidence rate (black line) and 95% CI (grey band) for new-onset type 1 diabetes among all Swedish residents under 30 between January 2007 and December 2023. The light grey shading indicates the months during which SARS-CoV-2 transmission occurred in Sweden

**Fig. 3:**
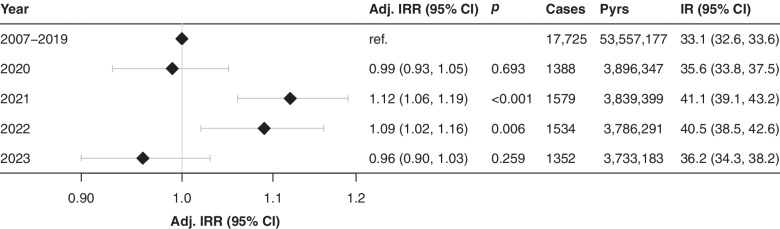
Adjusted IRR (adj. IRR) and 95% CI for each pandemic year compared with the pre-pandemic period (2007–2019). The IRR were derived using Poisson regression models adjusted for age, sex and the linear long-term annual trend. Incidence rates (IR) are given as cases per 100,000 person-years (pyrs) together with their 95% CI

**Fig. 4:**
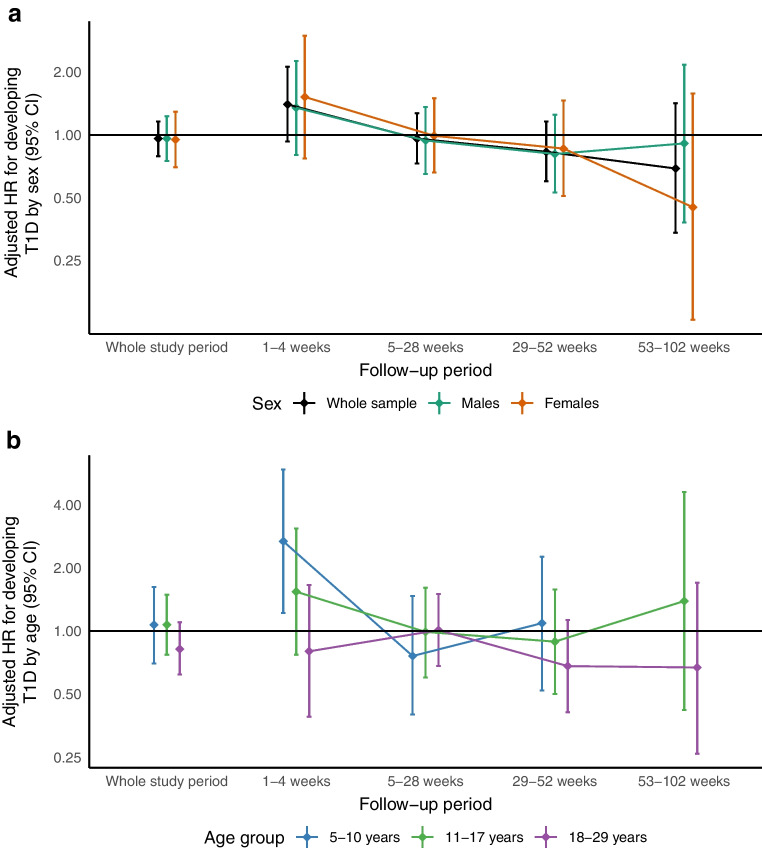
Adjusted HR for the risk of developing T1D in a cohort of individuals with confirmed SARS-CoV-2 infection together with up to five control individuals matched by age, sex, geographical region and case date, stratified by (**a**) sex and (**b**) age. The categories for the follow-up period correspond to the time since infection. HR and 95% CI were derived using Cox regression, adjusting for family history of T1D and T2D, disposable household income and vaccination status. There were too few cases among those aged 0–4 years, and among those aged 5–10 years after 53 weeks of follow-up, to estimate HR. T1D, type 1 diabetes; T2D, type 2 diabetes

All analyses were performed for the whole population, as well as groups stratified by age and sex. The age groups were chosen to represent pre-school age (0–4 years old), early school age (5–10 years old), teenage years (11–17 years old) and early adulthood (18–29 years old). To explore the time dependence of the HR (see Electronic supplementary material [ESM] Figs 1–4), we further stratified the follow-up period into the first 4 weeks, 5–28 weeks, 29–52 weeks and 53–102 weeks after infection in the Cox regression models. Analyses were performed using R version 4.4.0 (RStudio Inc., Boston, MA, http://www.rstudio.com/).

## Results

In total, there were 6,521,893 individuals under 30 years of age registered in Sweden at some point between 2007 and 2023 (‘incidence cohort’). The source population for the cohort study consisted of 4,251,210 individuals who were under 30 years old, lived in Sweden and were registered during the study period (1 February 2020 to 28 February 2022) (Fig. 1). Of these, 20,330 had a type 1 diabetes diagnosis before the start of the study period and residential information was unavailable for 56,366, leaving 4,174,514 individuals as eligible for the study. In total, there were 828,354 individuals with a confirmed SARS-CoV-2 infection and 1,921,868 individuals who had an infection in their household, either themselves or among their household members. After exclusion of cases that did not fulfil the inclusion criteria, 727,605 cases were matched with 3,535,503 control individuals (representing 1,900,104 unique individuals) in the ‘individual infection cohort’, and 1,918,168 cases were matched to 9,349,378 control individuals (3,108,053 unique individuals) in the ‘household infection cohort’. In the ‘individual infection cohort’, only a few COVID-19 cases were below 5 years of age (see Table 1 and ESM Figs 5 and 6). COVID-19 cases and control individuals were similar in terms of family history of diabetes, vaccination status and disposable household income (Table 1).

**Table 1:** Characteristics of individuals with a positive SARS-CoV-2 test (‘individual infection cohort’) or with a positive SARS-CoV-2 test themselves or among their household members (‘household infection cohort’) and their respective control individuals matched for age, sex, region and case date

	Individual infection cohort		Household infection cohort	
	COVID-19 cases	Control individuals	COVID-19 cases	Control individuals
*n*	727,605	3,535,503	1,918,168	9,349,378
Age group (years)				
0–4	7815 (1.1)	38,353 (1.1)	254,252 (13.3)	1,246,178 (13.3)
5–10	131,594 (18.1)	637,367 (18.0)	444,365 (23.2)	2,168,214 (23.2)
11–17	191,123 (26.3)	928,551 (26.3)	519,446 (27.1)	2,532,096 (27.1)
18–29	397,073 (54.6)	1,931,232 (54.6)	700,105 (36.5)	3,402,890 (36.4)
Female	372,059 (51.1)	1,802,840 (51.0)	938,156 (48.9)	4,561,827 (48.8)
Family history of T1D	10,451 (1.4)	45,533 (1.3)	30,733 (1.6)	136,424 (1.5)
Family history of T2D	34,889 (4.8)	162,179 (4.6)	107,552 (5.6)	465,570 (5.0)
Vaccinated (≥2 doses)^a^	173,833 (23.9)	783,901 (22.2)	342,582 (17.9)	1,531,938 (16.4)
Disposable household income (SEK)	4702 (3119–7519)	4702 (3035–6947)	5462 (3696–7734)	5373 (3437–7253)
T1D cases	156	658	504	2043
Follow-up time (person-years)	404,994	1,682,872	1,187,553	4,898,330
T1D incidence^b^	38.5 (32.7, 45.1)	39.1 (36.2, 42.2)	42.4 (38.8, 46.3)	41.7 (39.9, 43.6)

Values are *n* (%) for categorical variables and median (IQR) for continuous variables. For T1D incidence, 95% CI are provided. The follow-up period to calculate the T1D incidence starts with the respective SARS-CoV-2 case date and ends on the earliest of the date of T1D diagnosis, any subsequent SARS-CoV-2 infection, emigration, death or the end of the study (28 February 2022)

^a^At start of follow-up

^b^Per 100,000 person-years

SEK, Swedish krona; T1D, type 1 diabetes; T2D, type 2 diabetes

### Type 1 diabetes incidence trends between 2007 and 2023

The incidence of type 1 diabetes increased during the whole study period (Figs 2 and 3). The overall incidence per 100,000 person-years was 35.6 (95% CI 33.8, 37.5) in 2020; 41.1 (95% CI 39.1, 43.2) in 2021; 40.5 (95% CI 38.5, 42.6) in 2022; and 36.2 (95% CI 34.3, 38.2) in 2023 compared with 33.1 (95% CI 32.6, 33.6) during the pre-pandemic period between 2007 and 2019. Whereas the observed incidence in 2020 was in line with the predicted linear trend (incidence rate ratio [IRR] 0.99; 95% CI 0.93, 1.05), it increased by 12% in 2021 (IRR 1.12; 95% CI 1.06, 1.19) and 9% 2022 (IRR 1.09; 95% CI 1.02, 1.16). In 2023, the incidence reverted to pre-pandemic trends (IRR 0.96; 95% CI 0.90, 1.03).

The age-specific trends in type 1 diabetes incidence are shown in ESM Tables 1 and 2 and ESM Fig. 7. There was significant heterogeneity in the effect of the pandemic on type 1 diabetes incidence between the various age groups (*p*<0.001; log-likelihood ratio test comparing models with and without the interaction term between the pandemic year and age). Children up to 4 years old developed type 1 diabetes more often during 2021 and 2022 compared with what was expected based on the pre-pandemic (2007–2019) linear trend. In 2022, for example, the incidence was 48% higher in boys (IRR 1.48; 95% CI 1.20, 1.81) and that in girls was 41% higher (IRR; 1.41; 95% CI 1.11, 1.77) compared with the pre-pandemic linear trend (ESM Fig. 8). In 2023, the incidence reverted to pre-pandemic trends. No significant changes in type 1 diabetes incidence were seen among 5–10 year olds. For teenagers (11–17 years old), the incidence was lower in 2023 compared with the predicted linear trend, while among young adults (18–29 years old), the incidence only significantly increased among men and increased most significantly in 2021.

### Risk of type 1 diabetes after SARS-CoV-2 infection

The overall incidence of type 1 diabetes per 100,000 person-years during the study period was 38.5 (95% CI 32.7, 45.1) among SARS-CoV-2 positive cases and 39.1 (95% CI 36.2, 42.2) among the control individuals (Table 1) in the ‘individual infections cohort’. The adjusted HR for SARS-CoV-2 infection on the risk for developing type 1 diabetes during the complete follow-up period was 0.96 (95% CI 0.79, 1.16) in the ‘individual infections cohort’ (Fig. 4). The incidence of type 1 diabetes in the exposed group decreased with longer follow-up time from infection. Among all age groups, the HR decreased from 1.40 (95% CI 0.93, 2.12) during the first 4 weeks after infection to 0.96 (95% CI 0.73, 1.27), 0.83 (95% CI 0.60, 1.16) and 0.69 (95% CI 0.34, 1.24) 5–28 weeks, 29–52 weeks and 53–102 weeks after infection, respectively. Similar patterns were seen when stratifying by sex (Fig. 4a). An association between SARS-CoV-2 infections and incidence of type 1 diabetes was suggested among the children aged 5–10 years during the first 4 weeks after SARS-CoV-2 infection (Fig. 4b). During that time period, the HR for developing type 1 diabetes was 2.68 (95% CI 1.22, 5.89) among 5–10 year olds compared with 0.82 (95% CI 0.62, 1.10) among young adults between 18 and 29 years old. No associations were found beyond 4 weeks of follow-up in any age group.

The same patterns as above were generally seen in the sensitivity analyses using a test-negative design with individuals who tested negative for SARS-CoV-2 as control individuals (see ESM Table 3 and ESM Fig. 9). However, the effect size was lower. Among the whole sample and using the full follow-up period, the HR for developing type 1 diabetes was 0.77 (95% CI 0.56, 1.04).

**Fig. 5:**
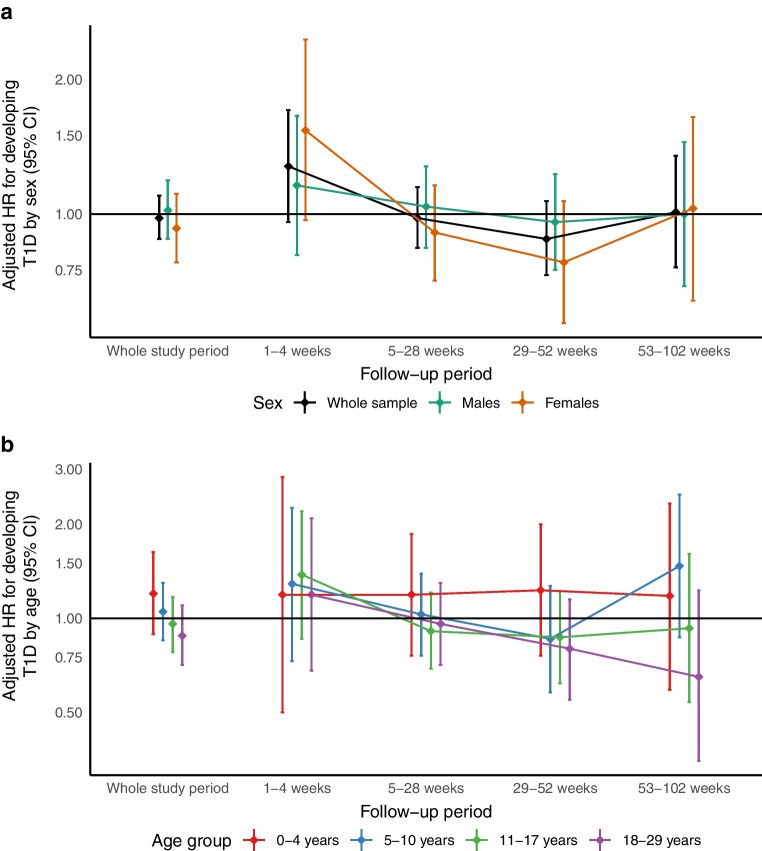
Adjusted HR for the risk of developing T1D in a cohort of individuals with confirmed SARS-CoV-2 infection themselves or among their household members. Cases were matched with up to five control individuals by age, sex, geographical region and case date. Estimates are stratified by (**a**) sex and (**b**) age. The categories for the follow-up period correspond to the time since infection. HR and 95% CI were derived using Cox regression, adjusting for family history of T1D and T2D, disposable household income and vaccination status. T1D, type 1 diabetes; T2D, type 2 diabetes

There were too few cases among the youngest age group (aged 0–4 years) to estimate the effect in the ‘individual infection cohort’. When using household infections as a proxy (the ‘household infection cohort’, comprising individuals with a COVID-19 case in the household), the same age trends were seen, but were not statistically significant (Fig. 5b). The overall IRR among children up to 4 years old was 1.20 (95% CI 0.89, 1.63).

## Discussion

Compared with the pre-pandemic linear trend, the incidence of type 1 diabetes among young individuals under 30 years old living in Sweden increased by 12% and 9% overall in 2021 and 2022, respectively, but reverted to pre-pandemic trends in 2023. The trends varied between age groups. The incidence among pre-school children (0–4 years old) only increased during 2021 and 2022, but it remained elevated until 2023 for young adult men (18–29 years old). No changes in type 1 diabetes incidence were seen among children between 5 and 10 years old. In 2023, the incidence among teenagers (11–17 years old) was even lower than expected based on the pre-pandemic linear trend. Analyses of two cohorts consisting of more than 700,000 individual COVID-19 cases as well as almost 2 million individuals who had an infection themselves or within their household showed no overall association between SARS-CoV-2 infections and the incidence of type 1 diabetes. However, children between 5 and 10 years old were more frequently diagnosed with type 1 diabetes during the first 28 days after SARS-CoV-2 infection. Nonetheless, the increases in type 1 diabetes among the total population appear not to be fully explained by a potential triggering effect of SARS-CoV-2 infections. Other risk factors that were affected during the pandemic probably contributed to these trends.

Increases in type 1 diabetes incidence have been observed in various countries across the globe [12, 23]. Our findings are in line with these trends, although no increases were seen during 2020, as in some other countries. A recent meta-analysis found similar increases of 13% in 2020 and 27% in 2021 compared with pre-pandemic years [12]. Similarly, the incidence of islet autoantibodies among children under 4 years old with a high genetic risk of developing type 1 diabetes was found to be increased during 2021 and 2022, but not in 2020 [24]. In 2023, the incidence returned to baseline trends in our study, consistent with trends in Scotland where the incidence had already returned to pre-pandemic levels by 2022 [25].

However, whether these increases are due to a potential triggering effect of SARS-CoV-2 infection on the aetiology of type 1 diabetes remains unclear. Even though there may be a physiological pathway through which COVID-19 may contribute to the development of type 1 diabetes and this association has been found in some studies [10, 13, 14, 16, 17, 26, 27], our results show no clear evidence that SARS-CoV-2 infections play a major role in the Swedish context. Our findings are thus in line with other studies in Finland and Denmark [9, 19, 22] as well as in other countries [20, 28] that did not find a clear contribution of SARS-CoV-2 infections to type 1 diabetes risk.

Nonetheless, two trends in our findings are noteworthy. First, the risk of developing type 1 diabetes after SARS-CoV-2 infection is higher with decreasing age, although this was not statistically significant. A similar age-dependency in the association between SARS-CoV-2 infections and type 1 diabetes was found in a large meta-analysis that included more than 11 million individuals [29]. Additional statistical power, potentially by conducting meta-analyses, may be necessary to ascertain associations among the youngest age group. Second, the risk in the first 4 weeks after SARS-CoV-2 infection was slightly increased, particularly among children between 5 and 10 years old. Such a pattern has been described in another study on SARS-CoV-2, although that study focused on adults [14]. This may indicate that infections accelerate the development of clinical symptoms, leading to a diagnosis among individuals who are living with type 1 diabetes in a pre-symptomatic stage, as has been observed for other viral infections [30]. However, the modest increases in type 1 diabetes risk in the first 4 weeks after SARS-CoV-2 infection among children between 5 and 10 years old cannot fully explain the increases in incidence among the total population during 2021 and 2022.

Potentially, factors other than SARS-CoV-2 infections may have contributed to the observed increase in type 1 incidence among the youngest age group during 2021 and 2022. In addition to a strong genetic component in the aetiology of type 1 diabetes, other environmental and lifestyle factors have been found to increase the risk of developing the disease [2, 4]. Many of these risk factors were affected by the pandemic. First, obesity, poor diet and low physical activity have been found to increase insulin resistance and hence contribute to the risk for developing type 1 diabetes (accelerator hypothesis) [2]. Lifestyle changes and associated risk factors for type 1 diabetes have been described among all age groups [31]. Sweden is an interesting case, since the country largely remained open during the pandemic, with national interventions primarily based on public health recommendations. During the pandemic, children may have reduced social contacts and outdoor activities due to these recommendations or anxiety over transmission risks. Indeed, a study on pre-school children in Sweden found increases in BMI among children between 3 and 4 years of age, potentially linked to poorer diets, increased screen time, and lower physical activity levels [32–34]. Second, according to the hygiene hypothesis, early exposure to other childhood infections can reduce the risk of developing autoimmune diseases such as type 1 diabetes [4]. Due to the reduced social contacts, the children may have been less exposed to such environmental triggers to the immune system. Indeed, infection rates among children decreased during the early phases of the pandemic among a large multi-national cohort of children who were at high genetic risk of developing type 1 diabetes [24]. It is conceivable that both hypotheses lead to delayed effects on type 1 diabetes incidence, which may explain why increases were not seen during the first year of the pandemic but only during 2021 and 2022.

Similarly, among young adults, the switch to remote education and the recommendation to work from home has increased screen time and promoted a more sedentary lifestyle [35]. During the pandemic, young people in Sweden were found to be less physically active and to have higher levels of obesity [33–36]. We also speculate that the persistently increased type 1 diabetes incidence among young adults may be an indication that these lifestyle changes persisted even after the pandemic. Together, these findings suggest that efforts to promote healthy lifestyles during public health emergencies remain crucial, regardless of the level of restrictions. To tailor such efforts, further research on potential differences between socioeconomic or occupational groups is necessary.

A particular strength of this study is the availability of extensive register data with complete population coverage that allows linking of family and household members, as well as the long follow-up duration (a 17-year period ending in 2023). This allowed us to assess trends after the period during which the pandemic was considered as a public health emergency. Furthermore, we were able to triangulate data from different registers to identify type 1 diabetes cases. A limitation of the study is the low testing rates among children, particularly among the youngest age groups. We therefore analysed two different cohorts comprising individual infections and infections among any household member, respectively. The former design is less prone to exposure misclassification among the cases, but some COVID-19 cases remain undetected and are potentially included as control individuals. The latter design allows us to include untested individuals who were infected with SARS-CoV-2 by a household member. However, some uninfected individuals may be misclassified as cases, and in both approaches, some control individuals were probably misclassified as unexposed to SARS-CoV-2. As a result, the estimates of the effect of SARS-CoV-2 infections on the risk of type 1 diabetes onset are likely to be biased towards the null. Furthermore, there may also be selection bias because of differences in testing behaviours. More health-conscious individuals or people with a perceived risk of adverse COVID-19 outcomes may be more likely to comply with public health recommendations, including testing. Nonetheless, we were able to adjust for family history of type 1 diabetes and type 2 diabetes and household income. Furthermore, our results are robust when applying a test-negative design.

In conclusion, our findings suggest that the increases in type 1 diabetes incidence in Sweden during the pandemic can only partially be attributed to a potential triggering effect of SARS-CoV-2 infections. These findings suggest that, even with a relatively mild public health response to the pandemic, other behavioural and lifestyle risk factors for developing type 1 diabetes may have contributed to the increased incidence. This calls for more research into how social and environmental factors influenced the type 1 diabetes risk during the pandemic in order to develop future intervention strategies.

## Supplementary Information

Below is the link to the electronic supplementary material.

Supplementary file1 (PDF 589 KB)

## Data Availability

The individual-level register data used in this study are not available for sharing.

## Abbreviations

IRR: Incidence rate ratio
SARS-CoV-2: Severe acute respiratory syndrome coronavirus 2

## Code availability

The code used for data management and analyses may be made available upon request by contacting the corresponding author.
