# Supplementary material for: Trends in type 1 diabetes incidence between 2007 and 2023 and their association with SARS-CoV-2 infection in a population-based matched cohort study among individuals under 30 years old in Sweden
Source: Diabetologia. 2025 Sep 18;68(12):2732–42. doi: 10.1007/s00125-025-06540-1 (PMC12594641; doi:10.1007/s00125-025-06540-1)
Supplement: Supplementary file 1 — Supplementary file1 (PDF 589 KB) [file 125_2025_6540_MOESM1_ESM.pdf]

ESM Table 1 T1D cases and incidence among females by age group and time period. Pyrs: person-years

Period	Age group	T1D cases	Pyrs	Incidence rate (IR)
2007-2019	0-4 years	1172	4082839.53	28.71 (27.09-30.4)
2007-2019	5-10 years	2286	4740704.07	48.22 (46.26-50.24)
2007-2019	11-17 years	1947	5857404.27	33.24 (31.78-34.75)
2007-2019	18-29 years	2362	11389704.9	20.74 (19.91-21.59)
2020	0-4 years	83	303348.167	27.36 (21.79-33.92)
2020	5-10 years	189	382540.074	49.41 (42.61-56.97)
2020	11-17 years	167	425916.704	39.21 (33.49-45.63)
2020	18-29 years	157	779477.309	20.14 (17.11-23.55)
2021	0-4 years	108	297964.91	36.25 (29.73-43.76)
2021	5-10 years	186	379647.819	48.99 (42.2-56.56)
2021	11-17 years	184	432115.83	42.58 (36.65-49.2)
2021	18-29 years	160	752782.323	21.25 (18.09-24.81)
2022	0-4 years	121	291798.389	41.47 (34.41-49.55)
2022	5-10 years	200	376895.553	53.07 (45.97-60.95)
2022	11-17 years	167	437926.359	38.13 (32.57-44.38)
2022	18-29 years	166	729455.904	22.76 (19.43-26.49)
2023	0-4 years	77	285631.868	26.96 (21.27-33.69)
2023	5-10 years	201	374143.288	53.72 (46.55-61.69)
2023	11-17 years	154	443736.888	34.71 (29.44-40.64)
2023	18-29 years	160	706129.485	22.66 (19.28-26.45)

ESM Table 2 T1D cases and incidence among males by age group and time period. Pyrs: person-years

Period	Age group	T1D cases	Pyrs	Incidence rate (IR)
2007-2019	0-4 years	1362	4328438	31.47 (29.82-33.18)
2007-2019	5-10 years	2311	5087975	45.42 (43.59-47.31)
2007-2019	11-17 years	2966	6253395	47.43 (45.74-49.17)
2007-2019	18-29 years	3319	11816715	28.09 (27.14-29.06)
2020	0-4 years	110	321590.8	34.2 (28.11-41.23)
2020	5-10 years	203	405280.1	50.09 (43.44-57.47)
2020	11-17 years	250	451577.5	55.36 (48.71-62.67)
2020	18-29 years	229	826616.5	27.7 (24.23-31.53)
2021	0-4 years	147	315026.4	46.66 (39.42-54.85)
2021	5-10 years	222	402937.1	55.1 (48.09-62.84)
2021	11-17 years	264	457959.2	57.65 (50.9-65.04)
2021	18-29 years	308	800965.5	38.45 (34.28-43)
2022	0-4 years	162	308034.3	52.59 (44.8-61.34)
2022	5-10 years	217	399664.3	54.3 (47.31-62.02)
2022	11-17 years	264	464012.9	56.89 (50.24-64.19)
2022	18-29 years	237	778503.3	30.44 (26.69-34.58)
2023	0-4 years	108	301042.3	35.88 (29.43-43.31)
2023	5-10 years	177	396391.6	44.65 (38.32-51.74)
2023	11-17 years	245	470066.5	52.12 (45.8-59.07)
2023	18-29 years	230	756041	30.42 (26.62-34.62)

ESM Table 3 Characteristics of individuals with a positive SARS-CoV-2 test ordered through the Swedish national health counselling hotline and controls matched for age, sex, region, and case date.

	Test-negative cohort	
	**Tested positive (cases)**	**Tested negative (control individuals)**
n	397,857	1,985,264
Age group (years)		
0-4	1564 (0.4)	7667 (0.4)
5-10	79,855 (20.1)	399,093 (20.1)
11-17	94,788 (23.8)	473,452 (23.8)
18-29	221,650 (55.7)	1,105,052 (55.7)
Female	203,772 (51.2)	1,017,003 (51.2)
Family history of T1D	5566 (1.4)	29,487 (1.5)
Family history of T2D	17,817 (4.5)	84,668 (4.3)
Vaccinated (≥2 doses; %)[a]	104,035 (26.1)	585,556 (29.5)
Disposable household income (SEK)	5318 (3183–7742)	5318 (3151–7748)

Values are n (%) for categorical variables and median (IQR) for continuous variables. The follow-up period to calculate the type-1-diabetes incidence starts with the respective SARS-CoV-2 case date and ends on the earliest of the date of type 1 diabetes diagnosis, any subsequent SARS-CoV-2 infection, emigration, death, or the end of the study (28 February, 2022).

[a]At start of follow-up

SEK=Swedish krona, IQR=interquartile range.

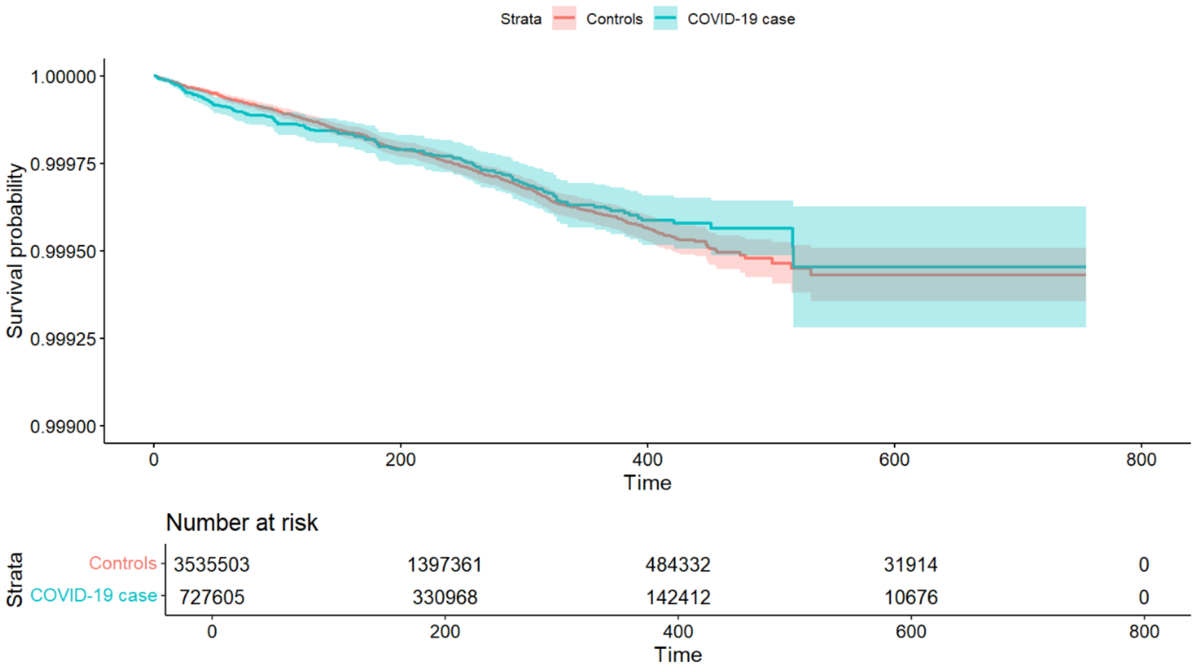

ESM Figure 1 Kaplan–Meier curves comparing COVID-19 cases with controls among the entire study population under 30 years. Individuals are followed from the day of SARS-CoV-2 infection until type 1 diagnosis, death, emigration, 30th birthday or the end of the study period (28 February 2022).

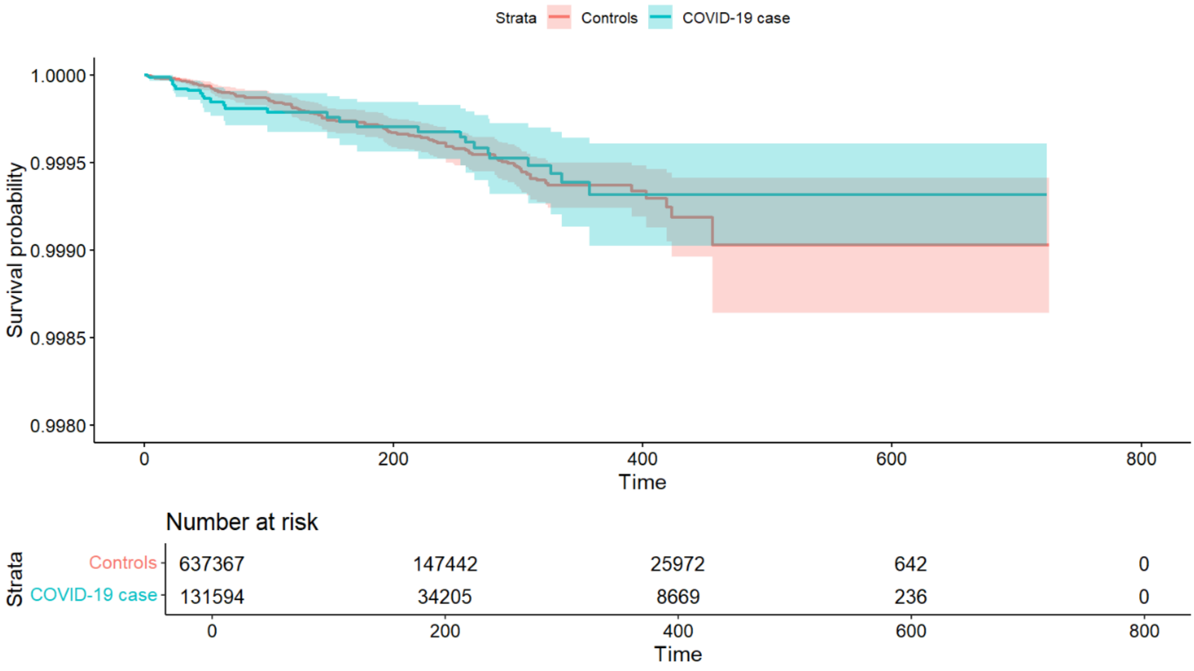

ESM Figure 2 Kaplan–Meier curves comparing COVID-19 cases with controls among children between 5 and 10 years. Individuals are followed from the day of SARS-CoV-2 infection until type 1 diagnosis, death, emigration, or the end of the study period (28 February 2022).

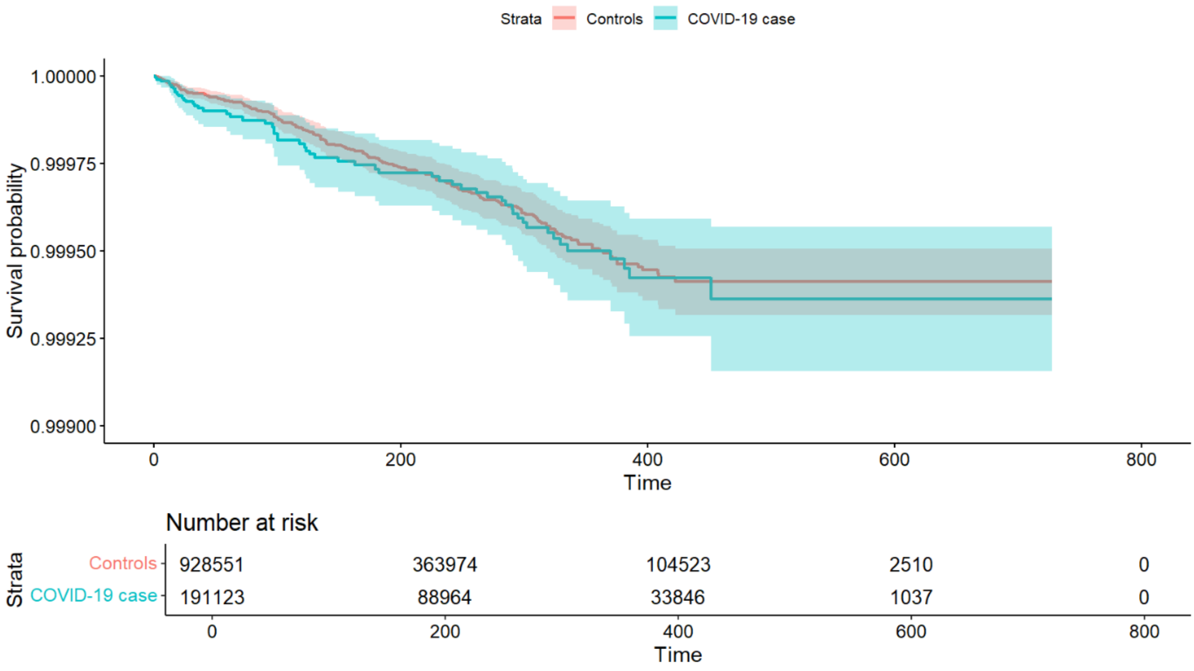

ESM Figure 3 Kaplan–Meier curves comparing COVID-19 cases with controls among teenagers between 11 and 17 years. Individuals are followed from the day of SARS-CoV-2 infection until type 1 diagnosis, death, emigration, or the end of the study period (28 February 2022).

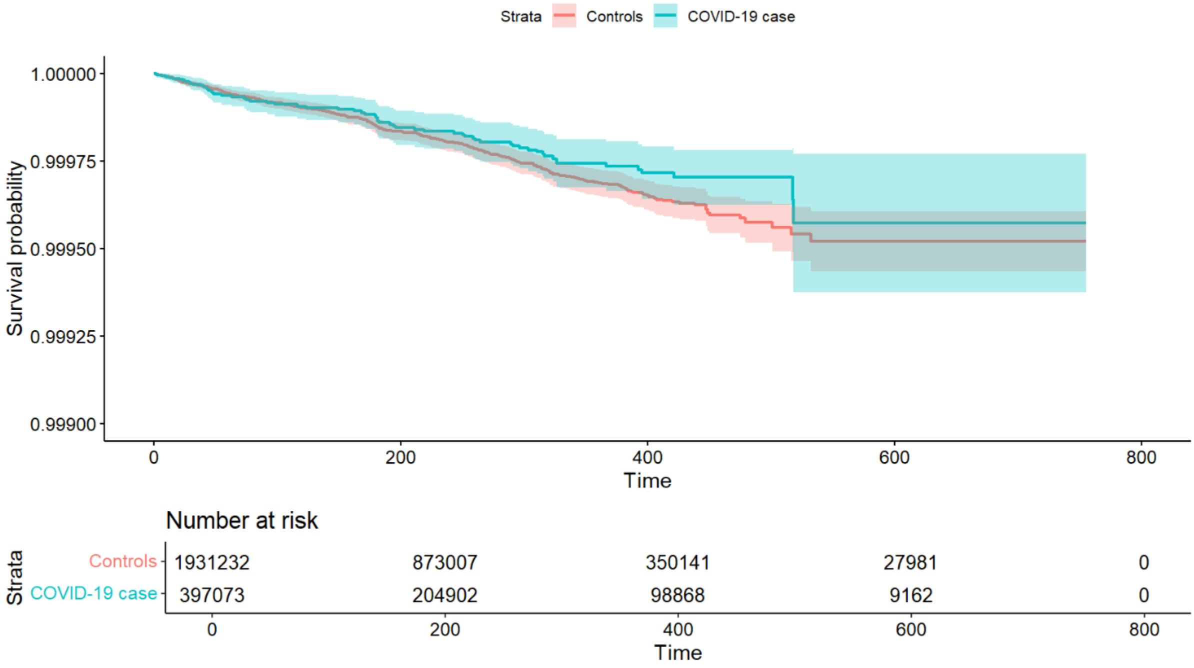

ESM Figure 4 Kaplan–Meier curves comparing COVID-19 cases with controls among young adults between 18 and 29 years. Individuals are followed from the day of SARS-CoV-2 infection until type 1 diagnosis, death, emigration, 30th birthday or the end of the study period (28 February 2022).

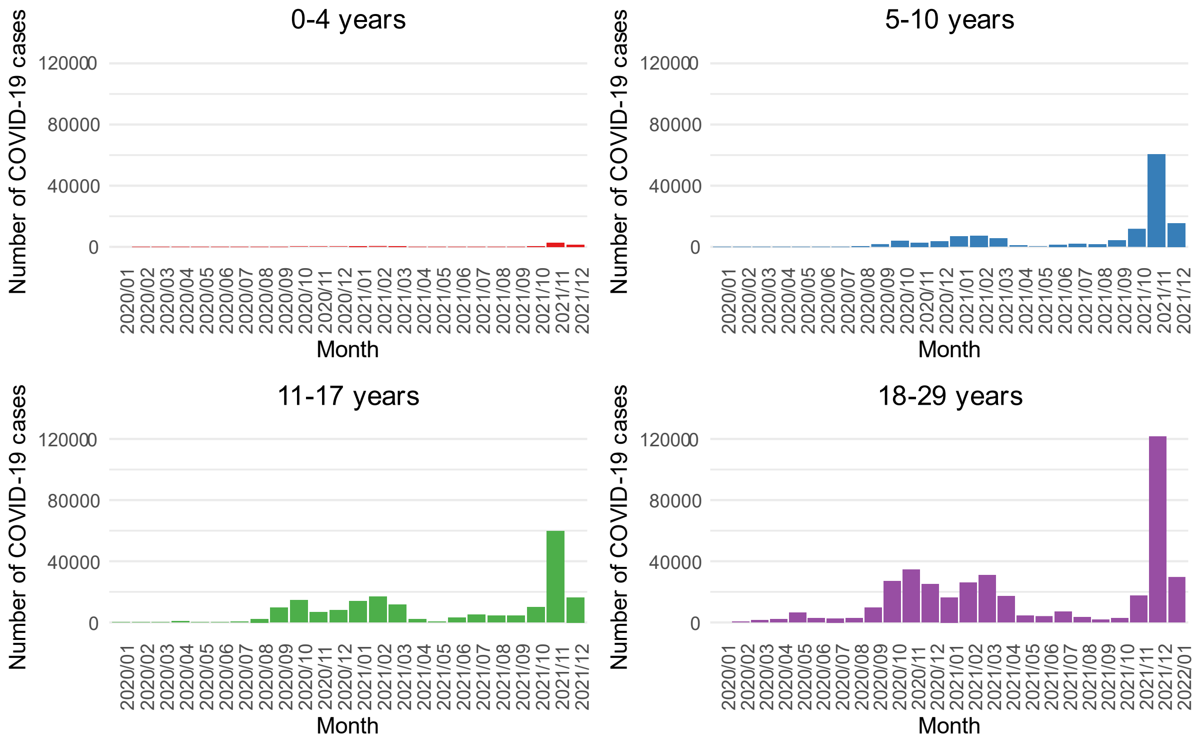

ESM Figure 5 Number of positive SARS-CoV-2 tests among individuals in the study population by age group and month.

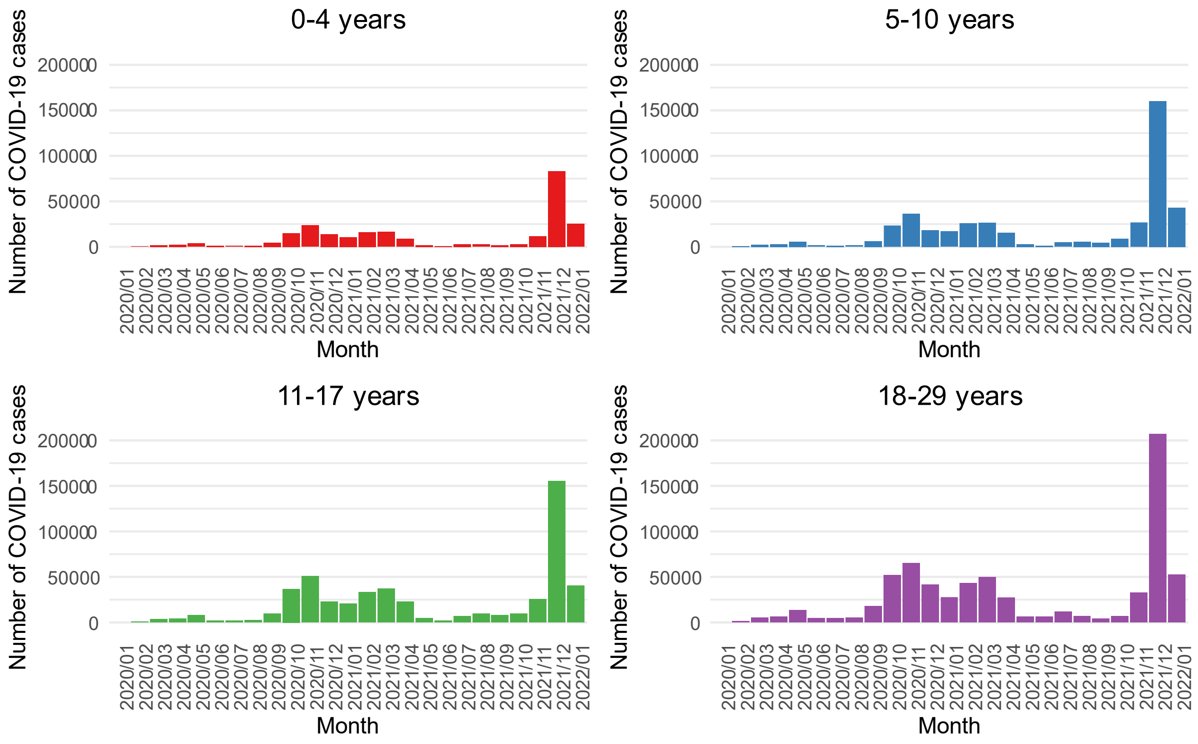

ESM Figure 6 Number of positive SARS-CoV-2 tests within the households of individuals in the study population by age group and month.

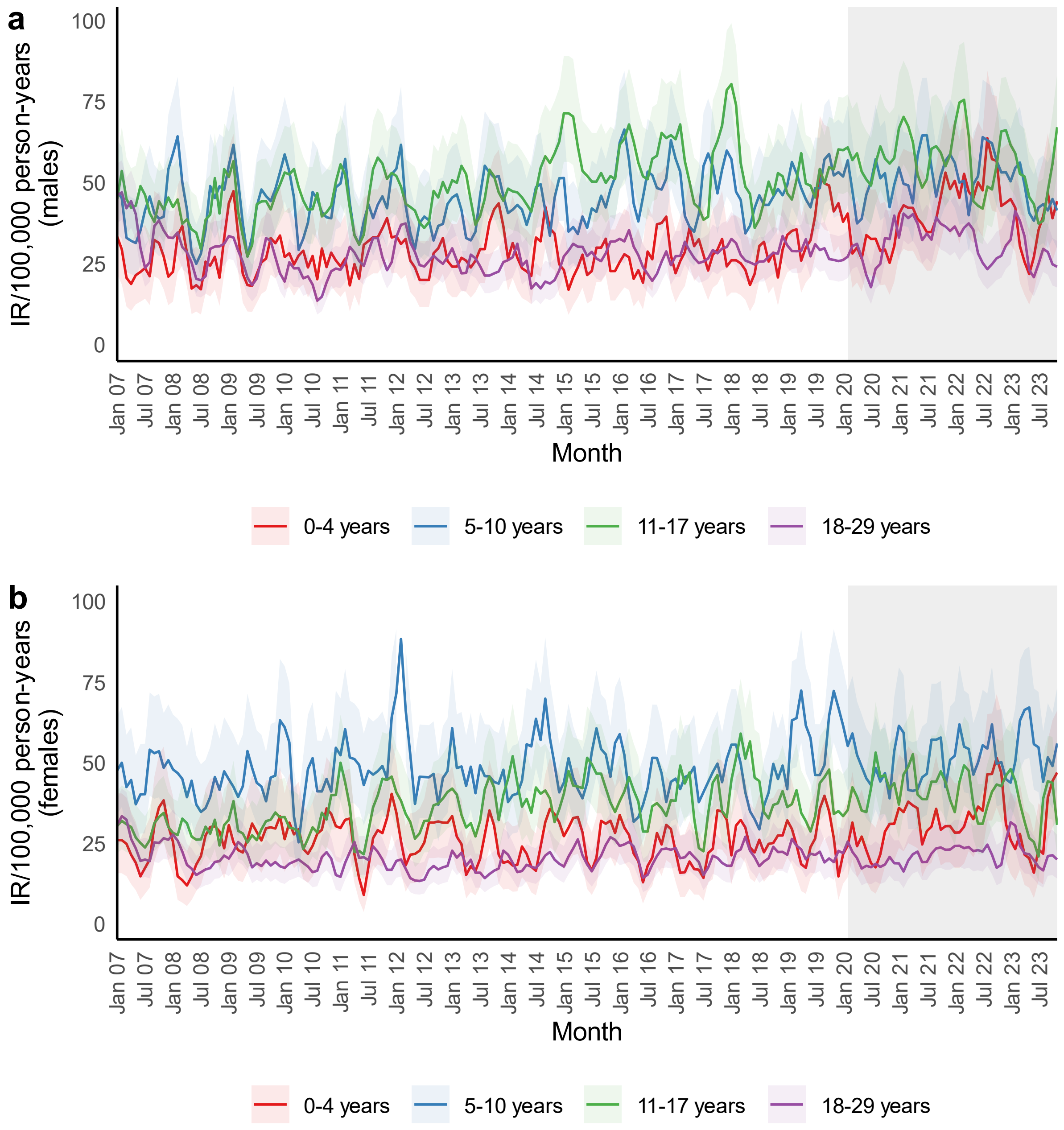

ESM Figure 7 Incidence rate (IR; solid lines) and 95% CI (coloured bands) for new-onset type 1 diabetes between January 2007 and December 2023 stratified by age group for (a) males and (b) females. The light grey shading indicates the months during which SARS-CoV-2 transmission occurred in Sweden.

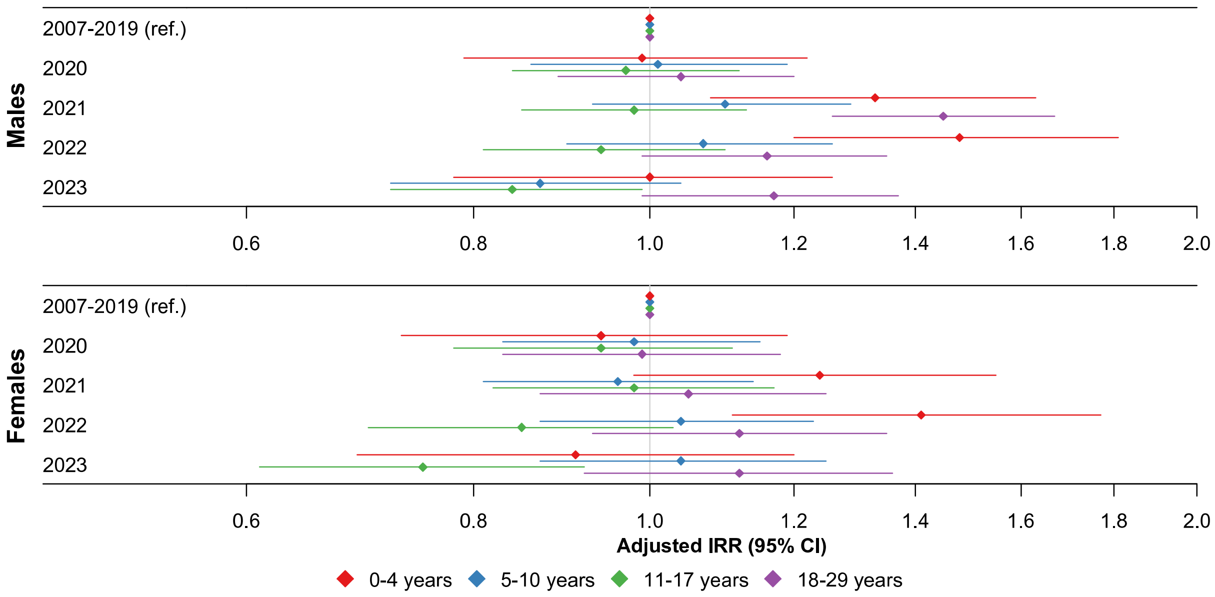

ESM Figure 8 Adjusted incidence rate ratio (adjusted IRR) and 95% CI for each pandemic year compared to the pre-pandemic period (2007-2019) stratified by age group among males (top) and females (bottom). The IRR were derived using Poisson regression models adjusted for the linear long-term annual trend.

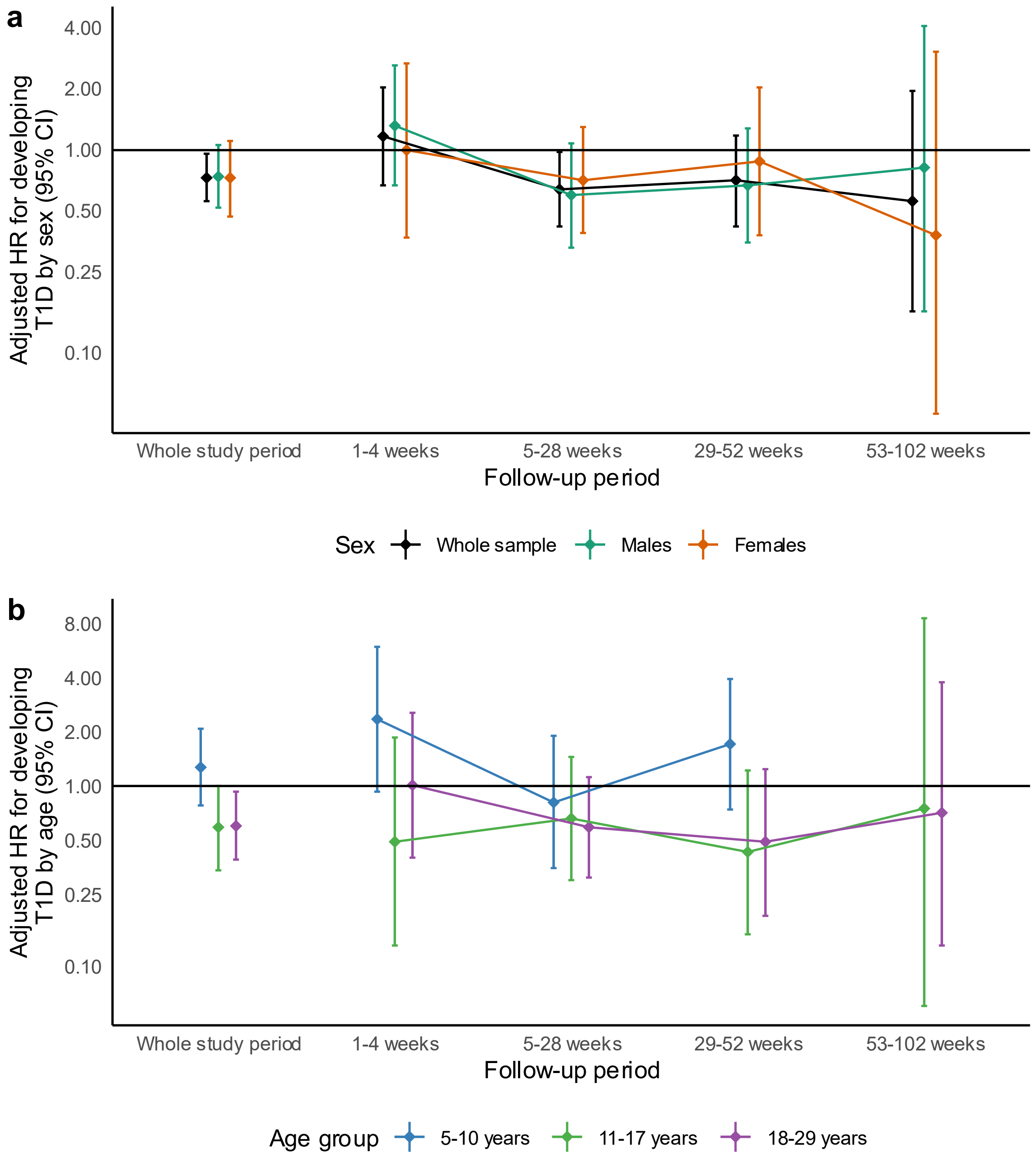

ESM Figure 9 Adjusted hazard ratio for the risk of developing T1D after SARS-CoV-2 infection in a test-negative cohort of individuals ordering SARS-CoV-2 tests through the Swedish health counseling service by (a) sex and (b) age group. Individuals tested positive for SARS-CoV-2 were matched with up to 5 controls that tested negative by age, sex, geographical region and test date (±14 days). HR and 95% CI were derived using Cox regression adjusting for family history of type 1 diabetes and type 2 diabetes, disposable household income, and vaccination status at case date.
